# High oral corticosteroid exposure and overuse of short-acting beta-2-agonists were associated with insufficient prescribing of controller medication: a nationwide electronic prescribing and dispensing database analysis

**DOI:** 10.1186/s13601-019-0286-3

**Published:** 2019-09-23

**Authors:** Ana Sá-Sousa, Rute Almeida, Ricardo Vicente, Nilton Nascimento, Henrique Martins, Alberto Freitas, João Almeida Fonseca

**Affiliations:** 1 CINTESIS-Center for Health Technology and Services Research, Faculdade de Medicina, Universidade do Porto, Porto, Portugal; 2 SPMS Shared Services of the Ministry of Health, Lisbon, Portugal; 3 MEDCIDS-Department of Community Medicine, Information, and Health Decision Sciences, Faculdade de Medicina, Universidade do Porto, Porto, Portugal; 4 Allergy Unit, Instituto & Hospital CUF Porto, Porto, Portugal

**Keywords:** Asthma, Pulmonary disease, chronic obstructive, Medication adherence, Inappropriate prescribing, Risk factors, Retrospective studies, Multivariate analysis

## Abstract

**Background:**

Recurrent use of oral corticosteroids (OCS) and over-use of short-acting beta-2-agonists (SABA) are factors associated with adverse side effects and asthma-related death. We aim to quantify high OCS exposure, SABA over-use and its association with prescription and adherence to maintenance treatment for respiratory disease, among patients with prescriptions for respiratory disease, from the Portuguese electronic prescription and dispensing database (BDNP).

**Methods:**

This was a 1-year (2016) retrospective population-based analysis of a random sample of adult patients from the BDNP, the nationwide compulsory medication prescription system. We assessed high OCS exposure (dispensing ≥ 4 packages containing 20 doses of 20 mg each of prednisolone-equivalent, ≥ 1600 mg/year) on patients on persistent respiratory treatment (PRT-prescription for > 2 packages of any respiratory maintenance medications). Excessive use of SABA was defined as having a ratio of SABA-to-maintenance treatment > 1 or having SABA over-use (dispensing of > 1 × 200 dose canister/month, of 100 μg of salbutamol-equivalent). Factors associated with high OCS exposure were assessed by multinomial logistic regression.

**Results:**

The estimated number of patients on PRT was 4786/100,000 patients. OCS was prescribed to more than 1/5 of the patients on PRT and 101/100,000 were exposed to a high-dose (≥ 1600 mg/year). SABA excessive use was found in 144/100,000 patients and SABA over-use in 24/100,000. About 1/6 of SABA over-users were not prescribed any controller medication and 7% of them had a ratio maintenance-to-total ≥ 70% (high prescription of maintenance treatment). Primary adherence (median%) to controller medication was 66.7% for PRT patients, 59.6% for patients exposed to high OCS dose and 75.0% for SABA over-users. High OCS exposure or SABA over-use were not associated with primary adherence. High OCS exposure was associated with a maintenance-to-total medication ratio < 70% (insufficient prescription of maintenance treatment), age > 45 years old and male sex.

**Conclusions:**

Exposure to high-dose of OCS (101 per 100,000 patients) and SABA over-use (24 per 100,000) were frequent, and were associated with a low maintenance-to-total prescription ratio but not with primary non-adherence. These results suggest there is a need for initiatives to reduce OCS and SABA inappropriate prescribing.

## Background

Chronic respiratory diseases, including obstructive lung diseases such as asthma and chronic obstructive pulmonary disease (COPD), are a source of significant morbidity and mortality worldwide [1]. The prevalence of asthma in adults in Portugal, in 2010, was 6.8% [2], costing 2.0% of the total Portuguese healthcare expense that year [3].

A report on asthma deaths in the United Kingdom highlights that most asthma deaths occur in mild and moderate cases of the disease, mainly because of inappropriate prescription and medical care [4]. According to Global Initiative for Asthma (GINA) guidelines, the factors that increase the risk of asthma-related death include (1) the over-use of inhaled short-acting beta2 agonists (SABA), defined as more than 1 canister of salbutamol or equivalent monthly; (2) the current use of oral corticosteroids (OCS); (3) the absence of inhaled corticosteroids (ICS) use and (4) the poor adherence with asthma maintenance medication [5]. Furthermore, exposure to OCS has been associated with pneumonia, osteoporosis, cataracts, and diabetes in a dose-responsive manner [6–8].

Large databases, including prescription data, has been used to assess the risk of asthma exacerbations [9, 10], describe patterns of frequent exacerbators [11] and inappropriate, high-risk prescriptions [12]. The use of pharmacy records, namely the number of SABA canisters filled over a 1-year period, has been validated as a proxy for future risk of (1) hospitalization or emergency department visit because of asthma and (2) OCS for exacerbations dispensing [13]. Observational studies based on medical records including prescription data are important to provide real-world evidence on heterogeneous diseases such as chronic respiratory diseases. However, to our knowledge, research based on Portuguese electronic prescription database is scarce [14, 15] and none on respiratory medication, namely OCS or SABA.

## Aims

We aim to quantify patients in high-risk of having adverse clinical outcomes, among patients with at least 1 prescription for respiratory disease or exacerbations medications, retrieved from the Portuguese electronic medical prescription and dispensing database. Specifically, we aim to describe the association of the exposure to high-dose of OCS and the SABA over-use with prescription and primary adherence to maintenance treatment for respiratory disease.

## Methods

### Study design

This was a 1-year (2016) retrospective population-based analysis of a random sample of patients from the BDNP—*Base de Dados Nacional de Prescrições* database.

### Setting

The BDNP is the central system, responsible for the validation of all steps of the prescriptions and dispensing cycle in Portugal, and for the recording of all the prescription and dispensing data. All the software for electronic medical prescription must be interoperable with BDNP. The use of electronic prescriptions is compulsory in mainland Portugal [16], and the system of electronic prescriptions is implemented since 2013. The electronic prescriptions system is used by physicians in the private and public healthcare units; each prescription may contain several medication packages and different classes of medication. The prescriptions must be filled at a community pharmacy by the patient. The implementation of the electronic medication dispensing system in each community pharmacy was concluded at the end of 2015.

**Fig. 1:**
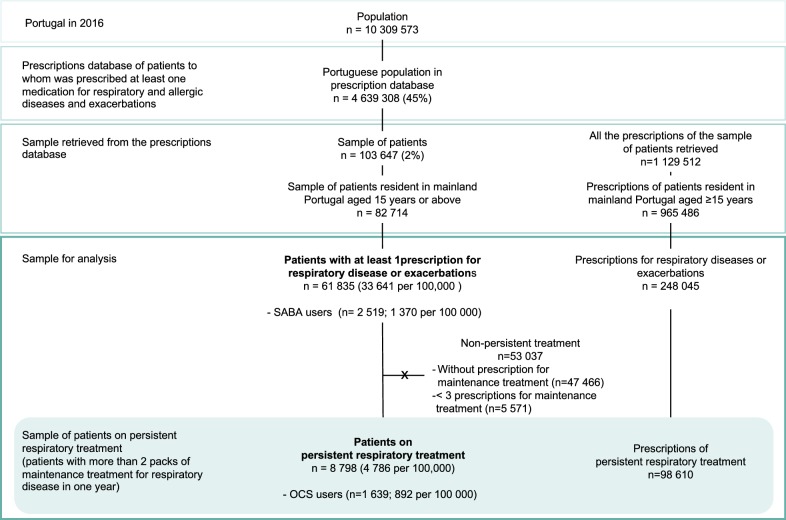
Flowchart of patients for analysis. *SABA* short-acting beta2 agonist, *OCS* oral corticosteroids

The population of interest in this study consists of patients to whom medication for respiratory and/or allergic diseases and exacerbations was prescribed at least once, between January 2016 and December 2016. The number of the prescriptions meeting these criteria was higher than to 250 million prescriptions, corresponding to 4,639,308 patients (45% of the Portuguese population). We retrieved 2% (n = 103 647) of these patients, randomly selected from the BDNP database corresponding to 1,129,512 prescriptions (Fig. 1). We assessed all the prescriptions of those aged 15 years old or above living in mainland Portugal (n = 82,714 patients). The number of patients in the sample per 100,000 Portuguese patients was calculated by multiplying the number of patients by the factor (45%/82,714).

Data were provided in an encrypted form by the government entity responsible for the electronic prescription and dispensing system, *SPMS*-*Serviços Partilhados do Ministério da Saúde* (Shared Services of the Ministry of Health). The data of the patients and the prescribing physician had previously been anonymised by SPMS.

### Participants

In this study we analysed the prescriptions (n = 248,045, corresponding to 61,835 patients) between January 2016 and December 2016 for medication for respiratory disease and/or exacerbations (see Additional file 1), from a sample of patients from the mainland Portugal, aged 15 years and above (Fig. 1).

### Variables

Persistent respiratory treatment (PRT) was defined as prescription for more than 2 packages of any of the six classes of respiratory maintenance medications: inhaled corticosteroids (ICS) alone or in fixed-dose combination with long-acting beta2 agonists (LABA); leukotriene receptors antagonists (LTRA); long-acting muscarinic antagonist (LAMA) alone or in a fixed-dose combination with LABA or LABA alone.

We analysed SABA usage in the sample of patients with at least one prescription for medication for respiratory disease and exacerbations whereas OCs usage was assessed only among patients on PRT, to reduce the confounding of its use for other conditions (Fig. 1).

OCS users and SABA users were defined as patients that filled, respectively, at least 1 package of OCS or SABA at a community pharmacy.

OCS dosage was estimated for OCS users, considering that 1 dose of OCS contains 5 mg of prednisolone or equivalent. SABA dosage was estimated for SABA users, considering that 1 dose contains 100 μg of salbutamol or equivalent. The total annual amount of prednisolone-equivalent and salbutamol-equivalent was estimated.

Considering that 1 package of prednisolone contains 400 mg of prednisolone (20 doses of 20 mg each), OCS annual amount of prednisolone-equivalent was grouped in low-dose (> 0:400 mg), medium dose (> 400:< 1600 mg) and high dose (≥ 1600 mg); corresponding to up to 1; > 1 to 3 and 4 or more packages of prednisolone [7].

The 1-year combinations of classes of respiratory maintenance treatment prescribed were assessed for each patient on PRT.

### Outcomes

OCS high-dose exposure: ≥ 4 packages (20 doses of 20 mg each) of prednisolone-equivalent, corresponding to ≥ 1600 mg of prednisolone-equivalent a year.

SABA over-use: > 1 canister (200 doses of 100 μg) of salbutamol-equivalent per month [5], corresponding to > 240,000 µg of salbutamol-equivalent a year.

Ratio SABA-to-maintenance: ratio of the packages of SABA filled over packages of maintenance treatment filled.

SABA excessive use was defined as having at least one: (1) SABA over-use or (2) ratio SABA-to-maintenance above 1:1.

Maintenance-to-total: percentage of the packages of maintenance treatment prescribed over the total (maintenance, relievers, and OCS) packages. This was dichotomized in < 70% and ≥ 70%. Insufficient prescription of maintenance treatment was considered for maintenance-to-total < 70% [17].

Primary adherence to controller medication: percentage of packages of maintenance medication filled over the packages prescribed. This was dichotomized in ≤ 50% and > 50% (medium adherence) and also in ≤ 70% and > 70% (high adherence), to explore its association with high OCS exposure and with SABA over-use.

### Statistical methods

Descriptive statistics were used to characterize the population and the maintenance treatment prescribed.

The association of OCS high-dose exposure was explored using multinomial logistic regression for age, sex, maintenance-to-total, excessive SABA use and primary adherence to controller medication (Additional file 3: Table S3). The predictors included in the final model were: age (grouped into 15–44; 45–64 and > 64 years old), sex and maintenance-to-total (dichotomized in < 70% and ≥ 70%). All analyses were performed using RStudio (Version 1.1.456^©^ 2009–2018 RStudio, Inc.). Adjusted odds ratios (OR) and 95% confidence intervals (CI) were reported for logistic regression results.

## Results

### Participants

In 2016, 33,640.9 per 100,000 patients were prescribed with at least 1 medication for respiratory diseases or exacerbations (Fig. 1), 17,450.2 per 100,000 with at least 1 medication for respiratory diseases and 16,190.7 per 100,000 with prescriptions for antibiotics, OCS or H1-antihistamines only.

Persistent respiratory treatment (PRT), defined as prescriptions for more than 2 packages of respiratory maintenance medications, was found in 4786.5 per 100,000 patients (Fig. 1). Patients’ characteristics are summarized in Table 1. Characteristics of the patients from the total sample are presented in Additional file 2.

**Table 1:** Characteristics of patients on SABA over-use, on PRT with high OCS exposure, and on PRT

	SABA over-use (23.9 per 100,000)		PRT with high OCS exposure (101.2 per 100,000)		PRT (4786.5 per 100,000)	
Sex (%) 95% CI						
Female	29.5	18.2–44.2	50.0	42.9–57.1	55.9	54.9–56.9
Male	70.5	55.8–81.8	50.0	42.9–57.1	44.1	43.1–45.1
Age, med P25–P75	61.0	50.8–73.5	69.0	57.3–78.8	64.0	47.0–76.0
Age (%) 95% CI						
15:44	11.4	5.0–24.0	8.1	4.9–12.9	28.8	27.9–29.8
45:64	45.5	31.7–59.9	30.1	24.0–37.0	21.8	20.9–22.6
> 64	43.2	29.7–57.8	61.8	54.7–68.5	49.4	48.3–50.4
Maintenance-to-total prescribed (%) 95% CI						
No controller prescribed	15.9	7.9–29.4	–		–	
> 0 to 20%	25.0	14.6–39.4	2.7	1.2–6.1	0.3	0.2–0.4
≥ 20 to < 50%	31.8	20.0–46.6	35.5	29.0–42.6	3.3	2.9–3.7
≥ 50 to < 70%	20.5	11.1–34.5	28.5	22.5–35.4	8.2	7.6–8.8
≥ 70 to < 90%	6.8	2.3–18.2	28.0	22.0–34.8	16.3	15.5–17.1
≥ 90 to 100%	0.0	0.0–8.0	5.4	2.9–9.6	72.0	71.0–72.9
Primary adherence to controller medication (%) 95% CI						
0%	10.8	4.3–24.7	3.8	1.8–7.6	6.9	6.4–7.4
> 0 to 20%	5.4	1.5–1.8	5.4	2.9–9.6	5.2	4.8–5.7
> 20 to 50%	13.5	5.9–27.9	31.7	25.4–38.7	28.2	27.3–29.1
> 50 to 70%	13.5	5.9–27.9	23.1	17.6–29.7	18.1	17.2–18.9
> 70 to 90%	35.1	21.8–51.2	21.0	15.7–27.4	19.4	18.6–20.2
> 90 to 100%	21.6	11.4–37.2	15.1	10.6–20.9	22.3	21.4–23.1

*PRT* persistent respiratory treatment, *SABA* short-acting beta2 agonist, *OCS* oral corticosteroids

### OCS usage

OCS was prescribed to 22.0% (95% CI 21.1–22.8) of the patients on PRT (1051.1 per 100,000) and dispensed to 18.6% (95% CI 17.8–19.5). Maintenance-to-total ratio of 70% or more was associated with a lower likelihood of having at least one dispensing of OCS (crude OR, 95% CI 0.2, 0.1–0.2) in patients on PRT.

**Fig. 2:**
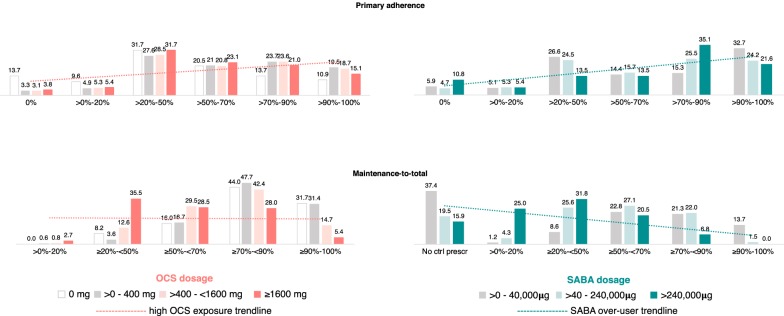
Frequency (%) of SABA users and OCS users on persistent respiratory treatment, by primary adherence to controller medication and ratio maintenance-to-total

Most of the OCS users were exposed to a low dose (> 0:400 mg) of prednisolone-equivalent (57.6%, 95% CI 55.2–60.6), still, 101.2 per 100,000 (11.3%, 95% CI 9.9–13.0) were exposed to a high-dose (≥ 1600 mg). Two-thirds of the patients exposed to high-dose of OCS had a ratio maintenance-to-total below 70% and 38.2% below 50% (Table 1 and Fig. 2).

### SABA usage

SABA was prescribed and dispensed to 1370.4 per 100,000 patients; 82.6% (95% CI 81.0–84.0) filled 2 or fewer canisters of salbutamol-equivalent, 15.7% (95% CI 14.3–17.1) filled 2 to 12 canisters and 1.7% (95% CI 1.3–2.3) were SABA over-users corresponding to 23.9 per 100,000 patients (Additional file 3). Excessive use of SABA (defined as SABA over-use or ratio SABA-to-maintenance above 1:1) was found in 10.5% of the SABA users, corresponding to 144.2 per 100,000 patients.

SABA over-users filled between 260,000 µg and 1,540,000 µg of salbutamol-equivalent, corresponding to a mean of 12 SABA inhalations per day per patient.

About 1/6 of the over-users were not prescribed any controller medication (Table 1). Among those with a prescription for maintenance treatment, 77% had maintenance-to-total below 70%, and 57% below 50% (Table 1 and Fig. 2).

### Primary adherence

In patients to whom maintenance treatment was prescribed, primary adherence to controller medication (median%, Percentile 25–Percentile 75) for the SABA over-users was 75.0% (47.6–88.9); for all PRT patients was 66.7% (33.3–87.5) and for the patients on PRT exposed to high-dose of OCS was 59.6% (37.5–82.9) (Table 1 and Fig. 2). Primary adherence to controller medication > 50% was not associated with reduced risk high OCS exposure nor with SABA over-use (OR, 95% CI 0.9, 0.7–1.2 and 1.4, 0.7–2.9, respectively). Similar results were observed for primary adherence to controller medication > 70% (OR, 95%; 1.4, 0.7–2.7 and 0.9, 0.7–1.2, respectively for SABA over-use and high OCS exposure).

### One-year maintenance treatment combinations

Among patients on PRT exposed to a high-dose (≥ 1600 mg) of OCS, the most frequent combinations of maintenance treatment were ICS + LABA or ICS + LABA + LAMA. The combinations ICS + LABA + LAMA; ICS + LTRA + LABA + LAMA or ICS + LTRA + LABA were found in 44% of these patients and monotherapy of either ICS or LTRA in 8% (Table 2).

**Table 2:** The 1-year combinations of classes of medication prescribed to the 8798 patients on PRT

Maintenance treatment prescribed	PRT with high OCS exposure (n = 186)		PRT	
	n	%	n	%
ICS + LABA	61	32.8	3113	35.4
ICS + LABA + LAMA	46	24.7	1008	11.5
ICS + LTRA + LABA + LAMA	21	11.3	355	4.0
ICS + LABA + LTRA	15	8.1	1204	13.7
LABA + LAMA	13	7.0	635	7.2
ICS monotherapy	8	4.3	310	3.5
LTRA monotherapy	6	3.2	916	10.4
LABA monotherapy	5	2.7	340	3.9
ICS + LAMA	3	1.6	143	1.6
LAMA monotherapy	3	1.6	476	5.4
ICS + LTRA	2	1.1	126	1.4
LTRA + LABA	1	0.5	50	0.6
LTRA + LAMA	1	0.5	41	0.5
ICS + LTRA + LAMA	1	0.5	22	0.3
LTRA + LABA + LAMA	0	0.0	59	0.7

*PRT* persistent respiratory treatment, *SABA* short-acting beta2 agonist, *OCS* oral corticosteroids, *ICS* inhaled corticosteroids, *LABA* long-acting beta2 agonists, *LTRA* leukotriene receptors antagonists, *LAMA* long-acting muscarinic antagonist

Most of the patients on PRT (61%) were prescribed for combinations of ICS + LABA, ICS + LABA + LTRA or ICS + LABA + LAMA (Table 2). Prescription of LTRA + LABA or/and LAMA, not recommended in the guidelines [5, 18], was prescribed to 2% of the patients.

### Factors associated with high OCS exposure

**Fig. 3:**
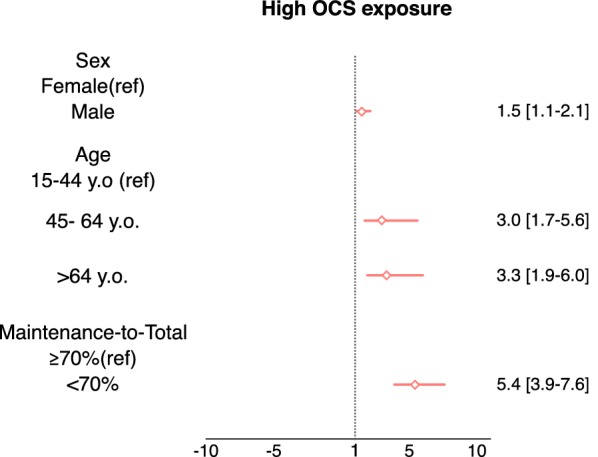
Factors associated (adjusted OR [95% CI]) to high OCS exposure

Results from the multinomial logistic regression, show that OCS high dose exposure was positively associated with maintenance-to-total < 70%, age above 45 years old and male sex (Fig. 3). The unadjusted independent associations of high OCS exposure are presented in Additional file 4.

## Discussion

### Limitations

This was the first analysis of prescriptions for SABA medication and OCS medication from the official Portuguese prescription database. Nevertheless, the present study has several limitations. An important limitation is related to the risk of overestimation of drug use since filling prescriptions does not mean actual medication intake. Another limitation is the lack of information regarding treatment indication. Moreover, OCS are prescribed for several conditions non-related to respiratory disease. In fact, some authors state OCS may not be a reliable marker of respiratory exacerbation [19]. To minimize this error, we analysed OCs usage only among patients on PRT (prescription for > 2 packages of any respiratory maintenance medications). Alternatively, we assessed OCS usage when ordered by prescribers with specialties related to respiratory disease and we obtained identical results (data not shown). In any case, SABA over-use and exposure to OCS are important risk-factors for serious adverse health outcomes, independently from the prescription indication.

The dataset has important limitations as it was not linked to non-prescription databases due to technical difficulties and privacy concerns, we could not assess the effect of important variables on the estimates, namely demographic variables [such as smoking habits, Body Mass Index (BMI), education, race].

**Fig. 4:**
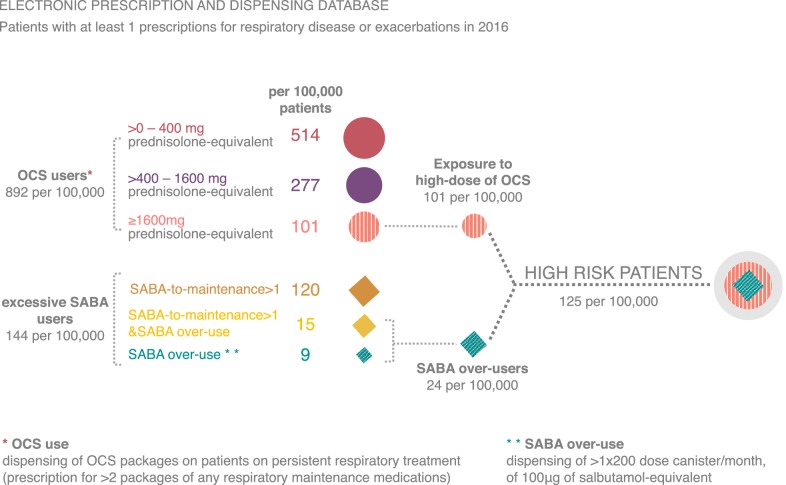
Patients with respiratory diseases with high-risk of having adverse clinical outcomes. *SABA* short-acting beta2 agonist, *OCS* oral corticosteroids

We used the Portuguese prescription and dispensing database to quantify patients with respiratory diseases with high OCS exposure or SABA over-use, that are associated with high-risk of having adverse clinical outcomes (Fig. 4). OCS use was assessed in the patients on persistent respiratory treatment (PRT) and high OCS exposure (≥ 1600 mg of prednisolone-equivalent) was found in 11.3% of the OCS users. Among SABA users, 10.5% were excessive users and 1.7% were SABA over-users. Patients on PRT with high OCS exposure and SABA over-users have primary adherence to controller medication above 50%. However, most of them have an insufficient prescription of maintenance treatment. Exposure to high-dose of OCS was associated with a ratio maintenance-to-total under 70%, male age above 45 years old and sex.

Almost 19% of patients on PRT dispensed at least 1 package of OCS and 11% of the OCS users dispensed at least 4 packages. Fitzgerald et al. [20] reported that 13% of asthma patients used OCS. Cumulative exposure to systemic corticosteroid is associated with adverse effects and substantial excess morbidity from multiple diseases [8, 21] and having 4 or more prescriptions of OCS per year has been shown to increase the incidence of adverse events in asthma patients [7].

In the present study, having a maintenance-to-total ratio below 70% was associated with the use of high-dose OCS. In our analysis we include prescriptions for respiratory patients and not only for asthma patients, therefore we used the ratio maintenance-to-total instead of the previously established ratios [17, 22, 23]. Although similar in its construct, the association of the ratio assessed in the present study with adverse outcomes may be different. We also observed that having a ratio of maintenance-to-total of 70% or more was associated with a lower likelihood of being OCS user. Accordingly, Stanford et al. [17] reported that controller-to-total asthma medication ratio of 70% or more, was associated with a reduction in OCS-dispensing events in 12-month follow-up (OR 0.81; 95% CI 0.76–0.88). In this study, the authors concluded that for adult Medicaid patients the optimal cut-off value was 70% and for the commercially insured patients was 50%. In Portugal in 2015, 65% of the health expenditures were supported by the government [24], as so we applied the cut-off of 70% to the ratio of maintenance-to-total, recommended for the Medicaid population. Nevertheless, since a ratio of less than 50% is known to be related to poor asthma control events, including the need for OCS [22, 25], we also tested the cut-off of 50% and found the maintenance-to-total of < 50% was associated with a higher likelihood of high OCS exposure (adj OR, 95% CI 7.6, 5.5–10.8).

The over-prescription of SABA with insufficient controller medication prescription remains frequent. In a 1-year study on asthma patients from primary care healthcare records, 6.6% of the SABA over-users were not on ICS [26]. We observed a higher rate (16%) of SABA over-users that did not receive a prescription for any controller medication in the 12-months period, a possible reason may be the analysis of all patients with respiratory treatment prescription and from all types of healthcare service, not only asthma patients from primary care. Moreover, in those with a prescription for controller medication, 77% had a ratio maintenance-to-total below 70%, and 57% below 50%. Overprescribing of SABA and insufficient provision of ICS was stated as a preventable cause of death for asthma [4] and other adverse outcomes, such as asthma-related hospitalizations, emergency department visits, and intense care unit admissions [20]. In agreement with the evidence on the risk of the use of SABA without any controller medication, the recently published pocket guide for asthma management by GINA network, recommends that ICS should be used whenever SABA is used, and ICS combined with formoterol may be used in low dose as the preferred reliever [27].

Our results suggest that high OCS exposure was associated with older age and male sex. Yang et al. [26] reported similar results for age but not for sex, as older age and female sex increased the risk of requirement for ≥ 2 courses of OCS for asthma exacerbations (adj OR, 95% CI 1.06, 1.01–1.12, for age and 0.64 0.45–0.89, for male sex), in a model adjusted also for SABA over-use (2.35, 1.42–3.89) and COPD (2.01, 1.34–3.01). Age and sex play important roles in the progression of chronic respiratory diseases. Aging of the airways and parenchyma induces structural and immunological changes related to the increase of airflow limitations. Reasons for gender differences may be related to anatomical, hormonal or socio-environmental factors [28].

Primary adherence was not associated with high-dose of OCS nor with SABA over-use. Previous studies show inconsistent results regarding the association between adherence to controller medication and SABA or OCS use. A systematic review indicated non-adherence as a risk factor for severe exacerbations, defined mostly as requiring for OCS, emergency department visit or hospitalization for asthma [29]. Makhinova et al. [30] shown that adherent patients, were more prone to have more than 6 prescriptions for SABA (OR 1.967, 95% CI 1.8–2.1), than nonadherent patients. Murphy et al. [31] reported that primary adherence to ICS below 80% was not associated with OCS courses, but was associated with the need for mechanical ventilation. A recent study on patterns of patients who experienced near-fatal asthma exacerbations reported that adherence to controllers may be an important factor for some patients (with rapid worsening of symptoms, young to middle-aged patients, smokers, with low BMI, tendency to depression and hypersensitive to environmental triggers), but not for other [32].

Of note, most of patients exposed to high-dose of OCS in our data were on a triple or quadruple combination of controller medication (ICS/LTRA + LABA + LAMA) associated with step 4/5 of treatment for asthma [5]. According to the guidelines these severe patients are possible candidates for treatment with monoclonal antibodies as in some severe phenotypes asthma remains uncontrolled despite good adherence to step 4/5 of treatment controller medication [33].

## Conclusion

OCS was prescribed to more than 1/5 of the patients on persistent respiratory treatment, and 101 per 100,000 patients were exposed to doses of OCS ≥ 1600 mg/year, associated with the risk of developing serious adverse outcomes. High OCS exposure was associated with a low maintenance-to-total prescription ratio, older age and male sex, but not associated with primary adherence to controller medication. Most SABA over-users had an insufficient prescription of maintenance treatment and about 1/6 were not prescribed for any maintenance medication, a known risk for asthma-related death. These results suggest a need for initiatives to reduce the number of high-risk patients with high OCS exposure and SABA over-use in Portugal.

## Supplementary information

**Additional file 1: Table S1.** Frequency of prescribed packages of medication for respiratory diseases and exacerbations.

**Additional file 2: Table S2.** Patients’ characteristics (n = 61,835).

**Additional file 3: Table S3.** Frequency of patients by number of SABA canisters dispensed in one-year period.

**Additional file 4: Table S4**. Association of sex, age, maintenance-to-total prescribed medication and primary adherence to high OCS exposure.

## Data Availability

The data that support the findings of this study are available from *Serviços Partilhados do Ministério da Saúde* (Shared Services of the Ministry of Health) but restrictions apply to the availability of these data, which were used under license for the current study, and so are not publicly available. Data are however available from the authors upon reasonable request and permission of *Serviços Partilhados do Ministério da Saúde* (Shared Services of the Ministry of Health).

## Abbreviations

BDNP: Portuguese electronic prescription and dispensing database
COPD: chronic obstructive pulmonary disease
GINA: Global Initiative for Asthma
ICS: inhaled corticosteroids
LABA: long-acting beta2 agonists
LAMA: long-acting muscarinic antagonist
LTRA: leukotriene receptors antagonists
OCS: oral corticosteroids
PRT: persistent respiratory treatment
SABA: short-acting beta-2-agonists
SAMA: short-acting muscarinic-antagonist
