# Supplementary material for: High oral corticosteroid exposure and overuse of short-acting beta-2-agonists were associated with insufficient prescribing of controller medication: a nationwide electronic prescribing and dispensing database analysis
Source: Clin Transl Allergy. 2019 Sep 23;9:47. doi: 10.1186/s13601-019-0286-3 (PMC6755705; doi:10.1186/s13601-019-0286-3)
Supplement: Supplementary file 1 — Additional file 1: Table S1. Frequency of prescribed packages of medication for respiratory diseases and exacerbations. [file 13601_2019_286_MOESM1_ESM.docx]

# Additional file 1

In this file we present the frequency of prescribed packages of medication for respiratory diseases and exacerbations, grouped by classes of medication.

Table S1: Frequency of prescribed packages of medication for respiratory diseases and exacerbations.

|  | Packages  (n=312 527) | |
| --- | --- | --- |
|  | n | % |
| Medication for respiratory diseases |  |  |
| Maintenance |  |  |
| ICS + LABA | 37 007 | 11.8 |
| LTRA | 21 085 | 6.7 |
| LAMA | 15 897 | 5.1 |
| LABA | 10 738 | 3.4 |
| ICS | 10 368 | 3.3 |
| LABA + LAMA | 8 051 | 2.6 |
| Relievers |  |  |
| SABA | 8 730 | 2.8 |
| SAMA | 5 639 | 1.8 |
| SABA + SAMA | 303 | 0.1 |
| Other |  |  |
| Expectorant (systemic) | 24 857 | 8.0 |
| Xanthine | 8 475 | 2.7 |
| Cough suppressant (systemic) | 4 691 | 1.5 |
| Cough suppressant with expectorant (systemic) | 81 | 0.0 |
| Anti Immunoglobulin E | 5 | 0.0 |
| Medication for exacerbation |  |  |
| Exacerbation/infection markers |  |  |
| Antibiotics | 55 810 | 17.9 |
| OCS | 27 399 | 8.8 |
| Other |  |  |
| H1-antihistamines (systemic) | 73 391 | 23.5 |

ICS: inhaled corticosteroids; LABA: long-acting beta2 agonists; LTRA: leukotriene receptors antagonists; LAMA: long‐acting muscarinic antagonist; SABA: short-acting beta 2 agonist; SAMA: Short-acting muscarinic-antagonist; OCS: oral corticosteroids.
