# Supplementary material for: High oral corticosteroid exposure and overuse of short-acting beta-2-agonists were associated with insufficient prescribing of controller medication: a nationwide electronic prescribing and dispensing database analysis
Source: Clin Transl Allergy. 2019 Sep 23;9:47. doi: 10.1186/s13601-019-0286-3 (PMC6755705; doi:10.1186/s13601-019-0286-3)
Supplement: Supplementary file 2 — Additional file 2: Table S2. Patients’ characteristics (n = 61,835). [file 13601_2019_286_MOESM2_ESM.docx]

# Additional file 2

In this file we present the characteristics of patients with at least 1 prescription for respiratory disease and exacerbations medications.

Table S2: Patients’ characteristics (n=61 835).

|  | Total | |
| --- | --- | --- |
| Sex, % 95%CI |  |  |
| Female | 60.7 | 60.3-61.1 |
| Male | 39.3 | 38.9-39.7 |
| Age, med P25-P75 | 53.0 | 37.0-69.0 |
| Age, % CI95% |  |  |
| 15:44 | 45.4 | 45.0-45.8 |
| 45:64 | 22.5 | 22.1-22.8 |
| >64 | 32.1 | 31.7-32.4 |
| Maintenance-to-total prescribed, % 95%CI |  |  |
| No control prescribed | 76.8 | 76.4-77.0 |
| >0%-20% | 0.2 | 0.2-0.2 |
| ≥20%-<50% | 1.0 | 1.0-1.1 |
| ≥50%-<70% | 2.9 | 2.8-3.0 |
| ≥70%-<90% | 2.3 | 2.2-2.4 |
| ≥90%-100% | 16.8 | 16.5-17.0 |
| Primary adherence to controller medication, % 95%CI |  |  |
| 0% | 13.9 | 13.3-14.4 |
| >0%-20% | 3.2 | 2.9-3.5 |
| >20%-50% | 19.8 | 19.1-20.5 |
| >50%-70% | 25.7 | 24.5-27.0 |
| >70%-90% | 27.6 | 26.3-28.9 |
| >90%-100% | 40.2 | 39.4-41.0 |
