# Supplementary material for: High oral corticosteroid exposure and overuse of short-acting beta-2-agonists were associated with insufficient prescribing of controller medication: a nationwide electronic prescribing and dispensing database analysis
Source: Clin Transl Allergy. 2019 Sep 23;9:47. doi: 10.1186/s13601-019-0286-3 (PMC6755705; doi:10.1186/s13601-019-0286-3)
Supplement: Supplementary file 3 — Additional file 3: Table S3. Frequency of patients by number of SABA canisters dispensed in one-year period. [file 13601_2019_286_MOESM3_ESM.docx]

# Additional file 3

In this file we present the distribution of SABA users by number of canisters dispensed in one-year period.

Table S3: Frequency of patients by SABA canisters dispensed in one-year period.

|  | Patients | |
| --- | --- | --- |
| SABA canisters* | n | % |
| ≤ 2 | 2080 | 82.6 |
| >2 - 4 | 213 | 8.5 |
| >4 - 8 | 126 | 5.0 |
| >8 - 12 | 56 | 2.2 |
| >12 | 44 | 1.7 |

*1 canister contains 200 doses of 100 μg of salbutamol-equivalent.
