# Supplementary material for: High oral corticosteroid exposure and overuse of short-acting beta-2-agonists were associated with insufficient prescribing of controller medication: a nationwide electronic prescribing and dispensing database analysis
Source: Clin Transl Allergy. 2019 Sep 23;9:47. doi: 10.1186/s13601-019-0286-3 (PMC6755705; doi:10.1186/s13601-019-0286-3)
Supplement: Supplementary file 4 — Additional file 4: Table S4. Association of sex, age, maintenance-to-total prescribed medication and primary adherence to high OCS exposure. [file 13601_2019_286_MOESM4_ESM.docx]

# Additional file 4

In this file we present the unadjusted associations with exposure to high-dose of OCS.

Table S4: Association of sex, age, maintenance-to-total prescribed medication and primary adherence to high OCS exposure

|  | Total OCS | | low/medium dose OCS | | high dose OCS | | p | Crude OR | |
| --- | --- | --- | --- | --- | --- | --- | --- | --- | --- |
|  |  |  |  |  |  |  |  | OR | 95%CI |
| Sex, n % |  |  |  |  |  |  | 0.03 |  |  |
| Female | 1119 | 57.9 | 1026 | 58.8 | 93 | 50.0 |  | 1 |  |
| Male | 813 | 42.1 | 720 | 41.2 | 93 | 50.0 |  | 1.4 | 1.1-1.9 |
| Age, n % |  |  |  |  |  |  | <0.001 |  |  |
| 15:44 | 384 | 19.9 | 369 | 21.1 | 15 | 8.1 |  | 1 |  |
| 45:64 | 559 | 28.9 | 503 | 28.8 | 56 | 30.1 |  | 2.7 | 1.6-5.1 |
| >64 | 989 | 51.2 | 874 | 50.1 | 115 | 61.8 |  | 3.2 | 1.9-5.8 |
| Maintenance-to-total prescribed, n % |  |  |  |  |  |  | <0.001 |  |  |
| >0%-20% | 15 | 0.8 | 10 | 0.6 | 5 | 2.7 |  | 1 |  |
| ≥20%-<50% | 188 | 9.7 | 122 | 7.0 | 66 | 35.5 |  | 1.1 | 0.4-3.6 |
| ≥50%-<70% | 408 | 21.1 | 355 | 20.3 | 53 | 28.5 |  | 0.3 | 0.1-1.0 |
| ≥70%-<90% | 847 | 43.8 | 795 | 45.5 | 52 | 28.0 |  | 0.1 | 0.0-0.4 |
| ≥90%-100% | 474 | 24.5 | 464 | 26.6 | 10 | 5.3 |  | 0.0 | 0.0-0.2 |
| Maintenance-to-total prescribed(2), n % |  |  |  |  |  |  | <0.001 |  |  |
| <70% | 611 | 31.6 | 487 | 27.9 | 124 | 66.7 |  | 5.2 | 3.8-7.2 |
| ≥70% | 1321 | 68.4 | 1259 | 72.1 | 62 | 33.3 |  | 1 |  |
| Excessive SABA use, n % |  |  |  |  |  |  | 0.3 |  |  |
| No | 1890 | 97.8 | 1710 | 97.9 | 180 | 96.8 |  | 1 |  |
| Yes | 42 | 2.2 | 36 | 2.1 | 6 | 3.2 |  | 1.6 | 0.6-3.4 |
| Primary adherence to controller medication, n % |  |  |  |  |  |  | 0.8 |  |  |
| 0% | 94 | 4.9 | 87 | 5.0 | 7 | 3.8 |  | 1 |  |
| >0%-20% | 111 | 5.7 | 101 | 5.8 | 10 | 5.4 |  | 1.2 | 0.5-3.5 |
| >20%-50% | 558 | 28.9 | 499 | 28.6 | 59 | 31.7 |  | 1.5 | 0.7-3.6 |
| >50%-70% | 407 | 21.1 | 364 | 20.8 | 43 | 23.1 |  | 1.5 | 0.7-3.7 |
| >70%-90% | 423 | 21.9 | 384 | 22.0 | 39 | 21.0 |  | 1.3 | 0.6-3.2 |
| >90%-100% | 339 | 17.5 | 311 | 17.8 | 28 | 15.1 |  | 1.1 | 0.5-2.9 |
| Primary adherence to controller medication, n % |  |  |  |  |  |  | 0.7 |  |  |
| ≤50% | 763 | 39.5 | 687 | 39.3 | 76 | 40.9 |  | 1 |  |
| >50% | 1169 | 60.5 | 1059 | 60.7 | 110 | 59.1 |  | 0.9 | 0.7-1.3 |
